# Integrating bioinformatics and machine learning analyses to identify immune-related secretory proteins and therapeutic small-molecule drugs in calcific aortic valve disease with type 2 diabetes

**DOI:** 10.3389/fimmu.2025.1634655

**Published:** 2025-10-08

**Authors:** Xiang Zhang, Jiahui Wang, Qian Hu, Bangyu Guo, Mengjie Hu, Xiaobo Yu, Shunbo Wei, Qiujie Luo, Yuqing Zhang, Shentao Li, Binhao Zhang, Caixia Gao, Shuang Wang, Jianliang Zhou

**Affiliations:** ^1^ Department of Cardiovascular Surgery, Zhongnan Hospital, Wuhan University, Wuhan, Hubei, China; ^2^ Hubei Provincial Engineering Research Center of Minimally Invasive Cardiovascular Surgery, Wuhan, Hubei, China; ^3^ Wuhan Clinical Research Center for Minimally Invasive Treatment of Structural Heart Disease, Wuhan, Hubei, China

**Keywords:** type 2 diabetes mellitus, calcific aortic valve disease, secretory proteins, bioinformatics, machine learning, immune infiltration

## Abstract

**Introduction:**

Type 2 diabetes mellitus (T2DM) is a globally prevalent metabolic disease, and emerging studies have revealed its strong association with calcific aortic valve disease (CAVD). Chronic inflammation, oxidative stress, and immune dysregulation induced by hyperglycemia in T2DM may accelerate CAVD progression, although the molecular mechanisms remain unclear.

**Methods:**

We integrated and analyzed four CAVD and two T2DM gene expression datasets from the GEO database. Through differential gene expression analysis, weighted gene co-expression network analysis (WGCNA), and secretory protein screening, we identified shared pathogenic genes between T2DM and CAVD. Protein-protein interaction (PPI) networks, functional enrichment analysis, and Connectivity Map (cMAP) prediction were conducted to identify potential therapeutic targets. A diagnostic model was constructed using 113 machine learning algorithms, and immune infiltration analysis was performed using CIBERSORT. The expression of key genes was validated in clinical valve tissue samples via RT-qPCR, Western blotting, and immunohistochemistry.

**Results:**

A total of 13 intersecting genes were identified as potential secretory biomarkers. The diagnostic model built with four key genes (CDH19, COL1A2, PRG4, and SPP1) showed excellent predictive performance (average AUC = 0.95). Immune infiltration analysis revealed significant differences in macrophage and T cell subtypes between CAVD and controls. CDH19 was downregulated, while COL1A2, PRG4, and SPP1 were significantly upregulated in T2DM-associated CAVD tissues. Among the candidate compounds, phorbol-12-myristate-13-acetate (PMA) emerged as a top therapeutic molecule potentially capable of reversing pathological gene expression.

**Conclusion:**

Our study identifies key secretory proteins and immune signatures in T2DM-associated CAVD and proposes a novel diagnostic model with strong clinical applicability. These findings offer new insights for early diagnosis and personalized treatment strategies in CAVD patients with T2DM.

## 1 Introduction

Type 2 diabetes mellitus (T2DM) represents a globally prevalent metabolic disorder characterized by escalating incidence rates paralleling lifestyle modifications and population aging. Beyond its hallmark disturbances in glucose homeostasis, T2DM is intricately linked to multifaceted pathophysiological processes encompassing chronic inflammation, oxidative stress, and dysregulated immune modulation (1, 2). These systemic perturbations markedly exacerbate cardiovascular morbidity, with calcific aortic valve disease (CAVD) emerging as a critical comorbidity. CAVD, a prevalent valvulopathy pathognomonically defined by progressive aortic valve fibrosis and calcification, culminates in aortic valve stenosis and predisposes to catastrophic cardiovascular sequelae, including heart failure and sudden cardiac death (3–5). Accumulating epidemiological evidence has demonstrated a significantly elevated CAVD prevalence in T2DM cohorts compared to non-diabetic populations, with T2DM independently predicting accelerated CAVD progression and adverse clinical outcomes (6, 7).

The mechanistic interplay between T2DM and CAVD pathogenesis is mediated through multiple secretory protein pathways. Chronic hyperglycemia in T2DM fosters a proinflammatory milieu characterized by persistent elevation of systemic pro-inflammatory cytokines, including IL-6, TNF-α and IL-1β (8, 9). These mediators not only perpetuate systemic inflammation but also potentiate localized inflammatory cascades within valvular interstitial cells, thereby accelerating fibrotic remodeling and osteogenic differentiation (10). Furthermore, oxidative stress—a cardinal metabolic derangement in T2DM—exacerbates CAVD progression through upregulation of extracellular matrix-degrading enzymes and profibrotic mediators such as TGF-β, collectively driving pathological matrix remodeling (11). Notably, immune dysregulation constitutes a pivotal pathogenic nexus between T2DM and CAVD. Recent investigations have identified aberrant activation patterns in monocyte-macrophage lineages among T2DM patients, with these immunocompetent cells secreting pro-inflammatory cytokines and proteolytic enzymes that synergistically promote valvular fibrocalcific transformation (12). Such immune-mediated mechanisms assume particular significance in CAVD pathogenesis, as T2DM-associated immune perturbations may critically accelerate valvular degeneration through feedforward inflammatory loops (13).

Given the pathophysiological nexus between T2DM and CAVD, the development of early diagnostic tools and personalized therapeutic strategies for T2DM patients assumes critical clinical urgency. To achieve early CAVD detection and timely intervention in high-risk populations, it is imperative to establish a comprehensive diagnostic framework incorporating novel biomarkers that reflect the intersecting pathomechanisms of T2DM and CAVD. Such a model, synergistically integrating multi-omics signatures of both disorders, holds dual potential: enhancing diagnostic precision at preclinical stages and informing mechanistically grounded therapeutic innovations.

In this study, we analyzed four CAVD datasets and two T2DM cohorts from the Gene Expression Omnibus (GEO) database using bioinformatics methods to identify T2DM-related hub genes and their mechanisms in CAVD. Potential therapeutic compounds for CAVD were also screened. Machine learning-based diagnostic models were constructed, with a four-gene panel (CDH19, COL1A2, PRG4, SPP1) showing optimal performance. The expression patterns of these genes were validated, and the model’s diagnostic efficacy was assessed using two independent CAVD cohorts from GEO. Finally, we investigated immune cell characteristics in CAVD to explore interactions between these genes and the immune system.

## 2 Methods

### 2.1 Microarray data acquisition and processing

Six raw expression datasets (GSE12644, GSE51472, GSE153555, GSE83453, GSE235995, GSE55492) comprising CAVD test/training cohorts, along with two T2DM datasets (GSE20966, GSE25724), were retrieved from the GEO database. Using the “sva” R package (v4.3.1) (14), batch correction was performed on four CAVD datasets (GSE12644, GSE51472, GSE153555, GSE83453) via the ComBat algorithm, generating an integrated CAVD expression matrix containing 44 calcified and 33 control samples. Datasets GSE235995 and GSE55492 were processed as independent test cohorts for subsequent validation. Detailed descriptive information of datasets was shown in **Table 1**.

**Table 1:** Descriptive statistics of the GEO datasets.

GEO accession	Platform	Origin	Sample control	CAVD	Species	Group
GSE12644	GPL570	Heart valve	10	10	Homo sapiens	Train group
GSE51472	GPL570	Heart valve	5	5	Homo sapiens	
GSE153555	GPL16791	Heart valve	10	20	Homo sapiens	
GSE83453	GPL10558	Heart valve	8	19	Homo sapiens	
GSE235995	GPL24676	Heart valve	4	5	Homo sapiens	Test group
GSE55492	GPL11154	Heart valve	10	9	Homo sapiens	
GEO accession	Platform	Origin	Sample Control	T2DM	Species	
GSE20966	GPL1352	Pancreatic tissue	10	10	Homo sapiens	
GSE25724	GPL6480	Pancreatic tissue	7	6	Homo sapiens	

CAVD,calcific aortic valve disease

T2DM, Type 2 diabetes mellitus

### 2.2 Differentially expressed genes analysis

DEGs were identified in the integrated CAVD and T2DM datasets using the “limma” R package (15), with thresholds set at adjusted p <0.05 and |log2FC| >0.585. Volcano plots and heatmaps were generated for DEG visualization.

### 2.3 WGCNA enrichment analysis of key genes

The WGCNA package (16) implemented in R was used to construct scale-free co-expression networks. Key steps included: (1) Median absolute deviation (MAD) filtering (genes with MAD = 0 excluded); (2) Sample quality control via “goodSamplesGenes”; (3) Network construction with soft threshold power β=5; (4) Module eigengene (ME) identification via principal component analysis; (5) Module-trait relationship assessment. Modules showing strongest positive/negative correlations with clinical traits were selected for downstream analysis.

### 2.4 Secretory protein gene extraction

A total of 3,947 secretory protein-coding genes were obtained from the “SPOCTOPUS predicted secreted proteins” class in the Human Protein Atlas (https://www.proteinatlas.org) (17).

### 2.5 PPI network construction

PPI networks were built using the STRING database (18) (confidence score >0.4) and visualized via Cytoscape v3.8.2. Top two modules identified by MCODE plugin were retained for further analysis.

### 2.6 Functional enrichment analysis

Gene Ontology (GO) and Kyoto Encyclopedia of Genes and Genomes (KEGG) pathway analyses were conducted using DAVID (19) with significance threshold p<0.05. Results were visualized as bubble plots and circos diagrams.

### 2.7 cMAP analysis

Upregulated genes from top PPI modules were input into cMAP (20) to identify potential CAVD therapeutics. The top 10 compounds with highest enrichment scores were selected.

### 2.8 Machine learning algorithms

Twelve algorithms (LASSO, Ridge, Stepglm, XGBoost, RF, Enet, plsRglm, GBM, Naive Bayes, LDA, glmBoost, SVM) were systematically evaluated across 113 combinatorial configurations (21). We employed a stacking ensemble strategy to integrate predictions from multiple base models. Specifically: Base models (12 algorithms) were trained using 10-fold cross-validation on the training set. Their predictions on the validation folds were used as meta-features. A generalized linear model (GLM) was trained as the meta-model on these meta-features. The final ensemble model was applied to the independent test sets (GSE235995 and GSE55492). The optimal model was defined by highest mean AUC.

### 2.9 Immune infiltration analysis

CIBERSORT quantified immune cell proportions in CAVD samples. Wilcoxon tests compared immune cell differences between calcified/control valves (p<0.05). Spearman correlation assessed biomarker-immune cell interactions (22).

### 2.10 Clinical sample collection

Calcified T2DM (n=3) and non-calcified control (n=3) aortic valves were obtained from Zhongnan Hospital of Wuhan University following ethical approval (Declaration of Helsinki). Informed consent was obtained preoperatively.

### 2.11 RNA isolation and RT-qPCR

Total RNA extracted with TRIzol^®^ (Invitrogen) was reverse-transcribed using PrimeScript™ RT Master Mix (Takara). RT-qPCR was performed on a 7500 Real-Time PCR System (Applied Biosystems) with TB Green Premix Ex Taq™ II (Takara) (23). Relative mRNA expression was calculated via 2-ΔΔCt method using GAPDH normalization.

### 2.12 Western blotting

Proteins isolated with RIPA buffer were separated on 10% SDS-PAGE gels, transferred to PVDF membranes, and probed with primary/secondary antibodies. Bands were visualized via ECL (New Cell & Molecular Biotech) and quantified using ImageJ v1.8 (24).

### 2.13 Immunohistochemistry

Paraffin-embedded valve sections (5 μm) were stained with anti-S100A8 (A12018, ABclonal) and S100A9 (A9842, ABclonal) antibodies. Staining areas were quantified using ImageJ under confocal microscopy (Olympus) (25).

### 2.14 Statistical analysis

All analyses were performed in R v4.2.0. Wilcoxon test compared two groups; Kruskal-Wallis test analyzed three groups. Survival analysis used log-rank test. p<0.05 indicated statistical significance.

### 2.15 Software and code availability

All statistical analyses and visualizations were performed using R software (v4.2.0). The main R packages used in this study include: sva (v3.48.0) for batch effect correction, limma (v3.56.0) for differential expression analysis, WGCNA (v1.72-5) for co-expression network construction, ggplot2 (v3.4.4) and pheatmap (v1.0.12) for plotting, caret (v6.0-94) and glmnet (v4.1-8) for machine learning model training and evaluation, and e1071 (v1.7-14) for Support Vector Machine implementation. Protein-protein interaction analysis was performed using the STRING database (v12.0) and visualized in Cytoscape (v3.8.2).

## 3 Results

### 3.1 Data processing

**Figure 1:**
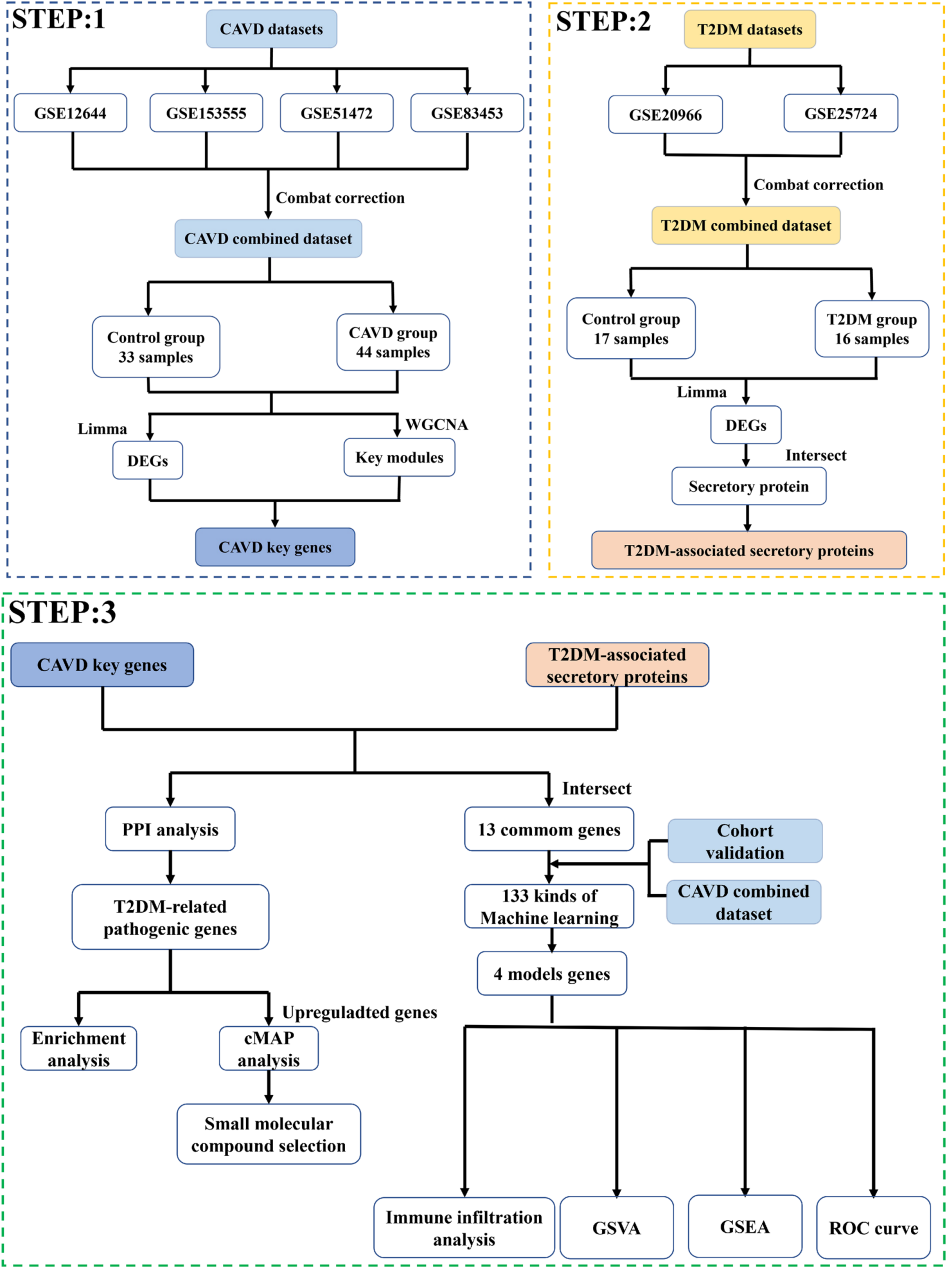
Research flow chart.

**Figure 2:**
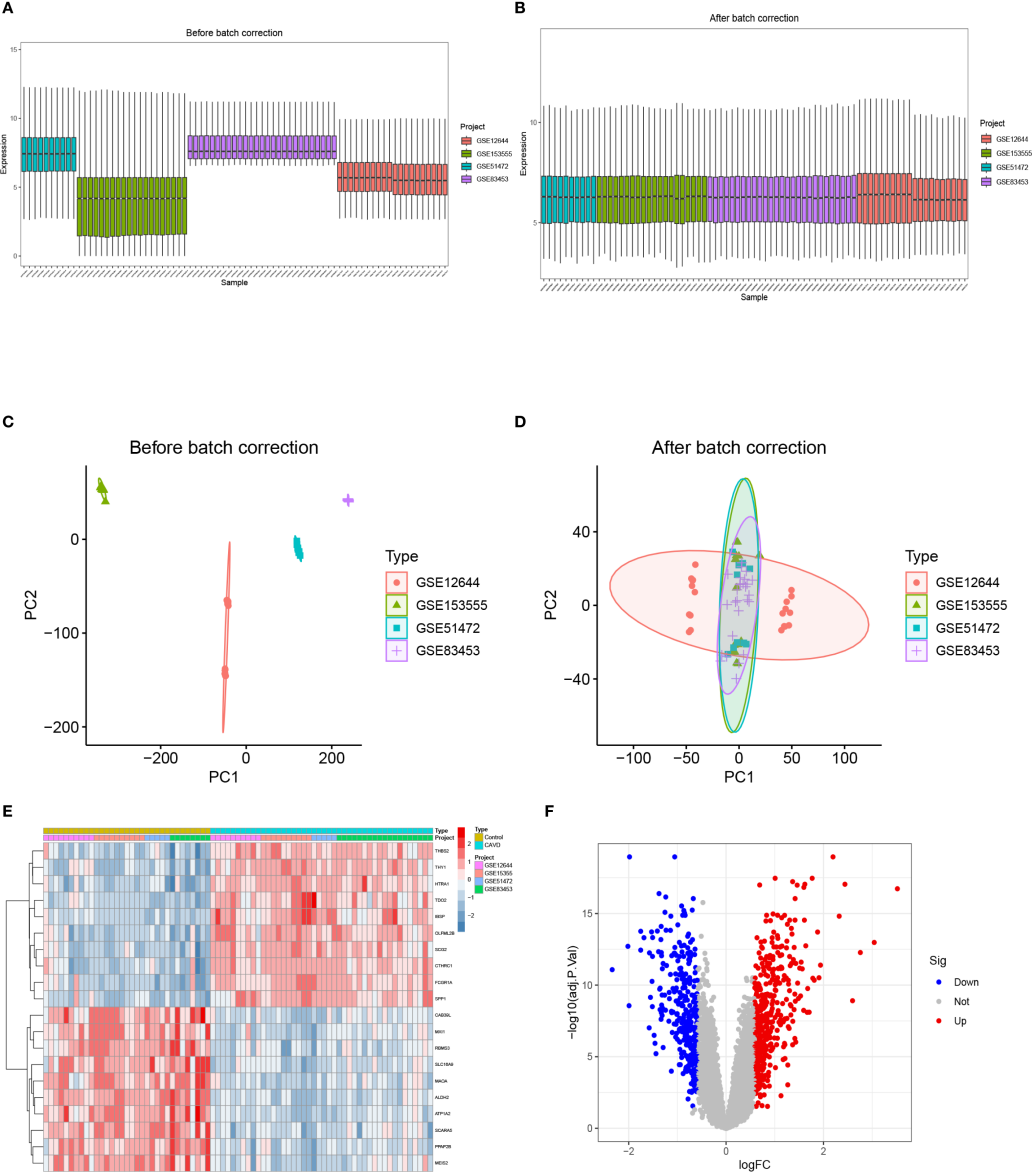
The integration of CAVD datasets and differential expression analysis of the integrated CAVD dataset. **(A)** Boxplot of raw data. **(B)** Boxplot after normalization. **(C)** PCA of three original CAVD datasets before batch-effect correction. **(D)** PCA of the integrated CAVD dataset after batch-effect correction. **(E)** The heatmap showing the top 30 upregulated and 30 downregulated DEGs in the integrated CAVD dataset. CAVD calcific aortic valve disease, PCA principal component analysis, DEGs differentially expressed genes. **(F)** The volcano plot representing CAVD DEGs in the integrated CAVD dataset. The upregulated genes are presented in red dots, whereas the downregulated genes are presented in blue dots.

The bioinformatics workflow is schematically depicted in **Figure 1**. Four raw aortic valve datasets (calcified vs. control samples) were retrieved from the GEO database and merged following batch effect correction using the “sva” package. Post-normalization, the integrated CAVD dataset comprised 44 calcified and 33 control samples. Principal component analysis (PCA) demonstrated significant reduction in inter-dataset heterogeneity after batch correction, as visualized in **Figures 2A–D**.

### 3.2 Identification of differentially expressed genes in calcific aortic valve disease

Comparative transcriptomic analysis between calcified and control aortic valve specimens identified 750 DEGs under predefined thresholds (adjusted p-value <0.05, |log2FC| >0.585), comprising 427 upregulated and 323 downregulated transcripts. The spatial distribution and hierarchical clustering patterns of these DEGs were visually represented through volcano plots and heatmaps, as illustrated in **Figures 2E, F**.

### 3.3 Construction of weighted gene co-expression network and identification of key modules in CAVD

**Figure 3:**
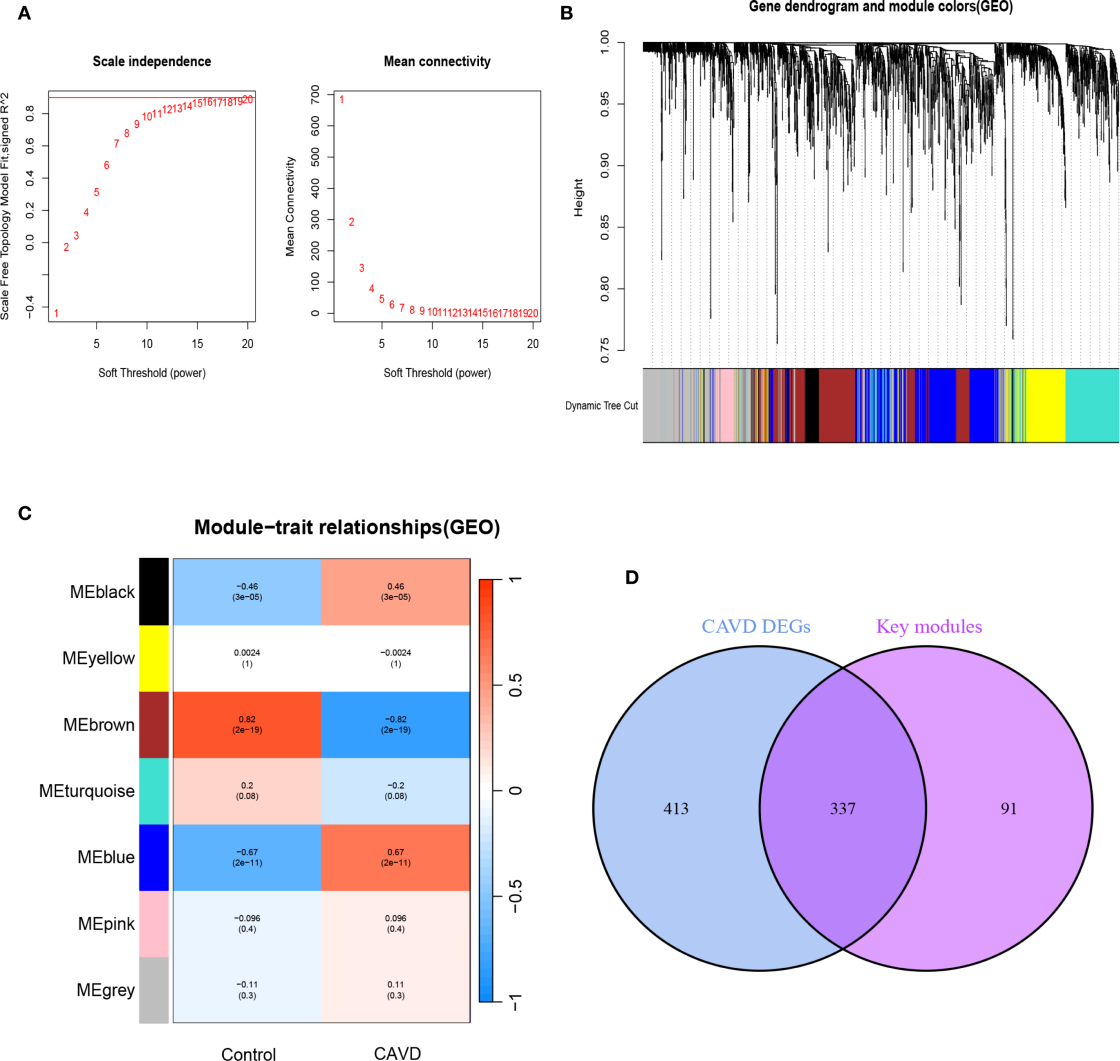
Screening of key module genes in the integrated CAVD dataset via WGCNA and identification of CAVD key genes through the intersection of key module genes and DEGs. **(A)** The scale-free topology model was utilized to identify the best β value, and β = 5 was chosen as the soft threshold based on the average connectivity and scale independence. **(B)** The network heatmap showing the gene dendrogram and module eigengenes. **(C)** The heatmap revealing the relationship between module eigengenes and status of CAVD. The correlation (upper) and *p*-value (bottom) of module eigengenes and status of CAVD were presented. The pink and yellow modules correlated with CAVD exhibited the highest and lowest correlation coefficients, respectively, which were identified as the key modules in CAVD. **(D)** A total of 337 key genes in CAVD were identified by taking the intersection between key modules genes and DEGs via the Venn diagram. WGCNA weighted gene co-expression network analysis, CAVD calcific aortic valve disease, DEG differentially expressed genes.

To delineate pivotal genetic determinants in CAVD pathogenesis, a WGCNA was implemented. Scale-free network construction employed soft threshold power β=6, determined through scale-free topology fit (R²>0.85) and mean connectivity optimization (**Figure 3A**). Hierarchical clustering dendrogram partitioned co-expressed genes into seven discrete modules, with topological relationships visualized through module eigengene clustering (**Figure 3B**). Module-trait correlation analysis revealed the brown module as most significantly associated with CAVD pathogenesis, encompassing 429 genes demonstrating strong positive correlation (r=0.82, p= 2e-19) with disease phenotype (**Figure 3C**). Intersectional analysis between CAVD-associated DEGs and WGCNA-derived hub genes identified 337 consensus candidates (**Figure 3D**), prioritized for subsequent functional investigations.

### 3.4 Identification of differentially expressed secretory proteins in type 2 diabetes mellitus

**Figure 4:**
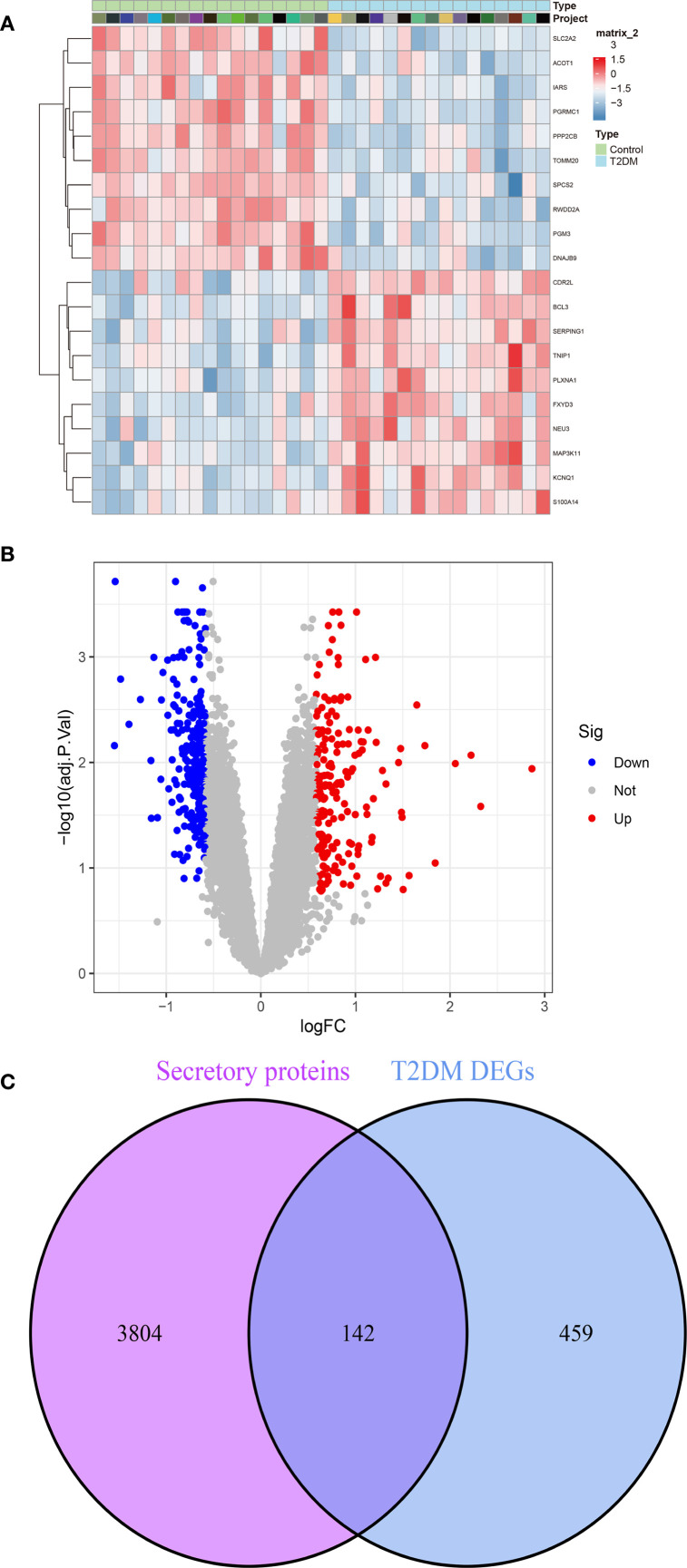
Identification of T2DM-associated secretory proteins through differential expression analysis in pancreatic tissues. **(A)** Heatmap depicting the top 10 upregulated (red) and downregulated (blue) DEGs in T2DM datasets. **(B)** Volcano plot visualizing DEGs in T2DM **(C)** Venn diagram illustrating the intersection between T2DM DEGs and secretory protein-coding genes, identifying 142 T2DM-associated secretory proteins.

Evidence from prior studies establishes a causal relationship between T2DM and accelerated CAVD progression. To investigate T2DM-associated pathogenic mechanisms in CAVD, we conducted systematic reanalysis of T2DM expression profiles from the GEO database. Volcano plot and heatmap visualization delineated 445 DEGs in T2DM (p<0.05, |log2FC|>0.585). (**Figures 4A, B**). Given the postulated secretory protein-mediated mechanism underlying T2DM-CAVD pathogenesis, intersectional analysis between T2DM DEGs and curated secretory protein genes identified 142 T2DM-associated differentially expressed secretory proteins (**Figure 4C**).

### 3.5 Functional enrichment analysis of T2DM-associated pathogenic genes in CAVD via PPI network screening

**Figure 5:**
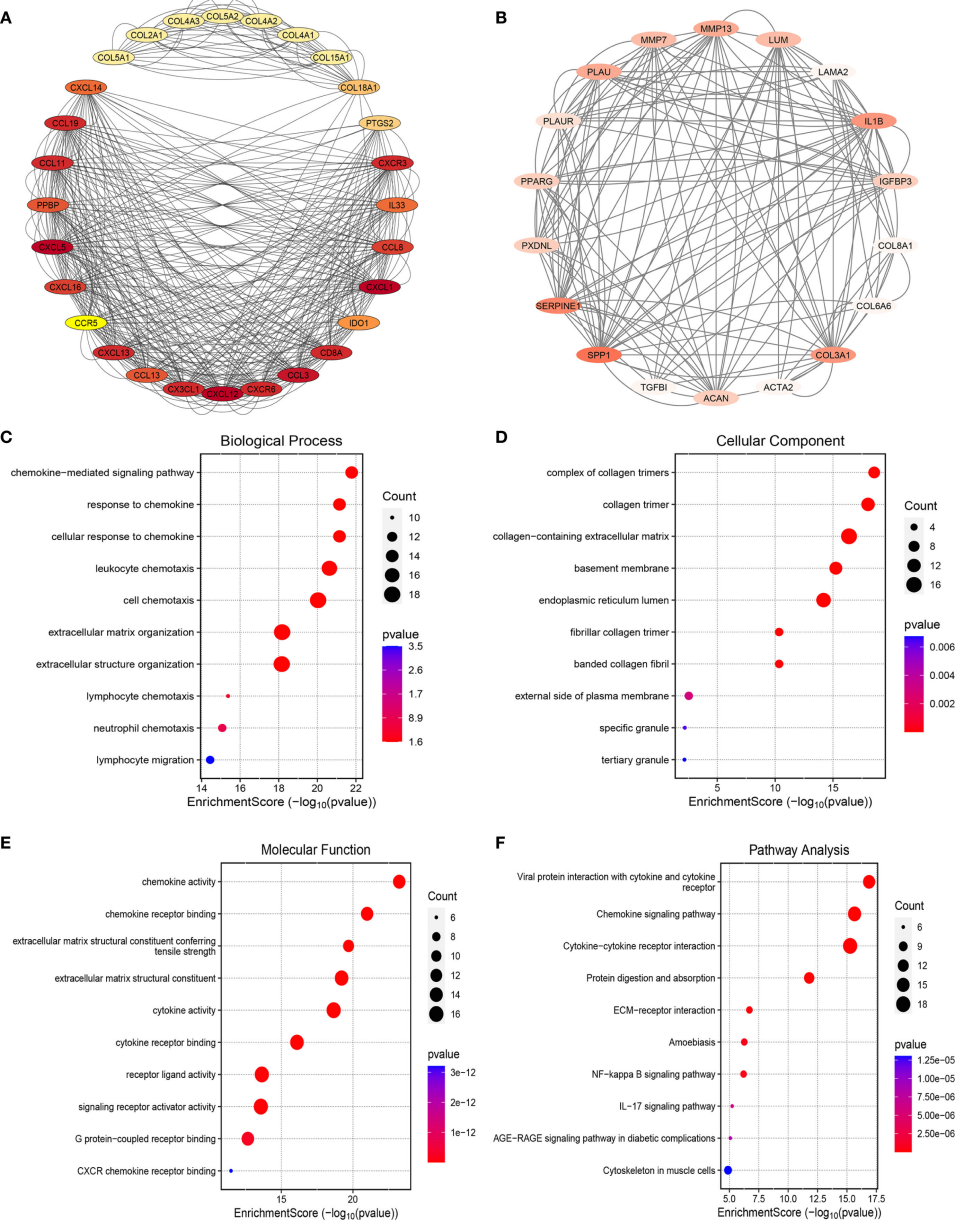
PPI analysis between T2DM-associated secretory proteins and CAVD key genes and followed by enrichment analysis of the PPI-screened nodes. **(A)** The PPI network of module1 genes with the top1 highest score based on Cytoscape plug-in MCODE analysis. Salmon nodes are marked as members of CAVD key genes, yellow nodes as members of T2DM-associated secretory proteins, while red nodes as common genes of the two sets. **(B)** The PPI network of module2 genes with the top2 highest score according to MCODE analysis. (**C**–**F)** The bubble plots showing the GO enrichment analysis results, including biological process **(C)**, cellular component **(D)**, and molecular function **(E)** of genes included in module1 and module2. **(F)** Circos plot representing the KEGG analysis results of genes included in module1 and module2. PPI protein-protein interaction, T2DM chronic kidney disease, CAVD calcific aortic valve disease, MCODE molecular complex detection.

To elucidate the molecular mechanisms underlying T2DM-related CAVD pathogenesis, we constructed a PPI network using the STRING database (interaction confidence score >0.4), integrating T2DM-associated secretory proteins and CAVD hub genes. Cytoscape visualization and MCODE clustering identified two critical modules containing 46 T2DM-associated pathogenic genes (**Figures 5A, B**). Functional enrichment analysis of these genes revealed significant involvement in chemokine-related pathways. GO analysis demonstrated enrichment in biological processes such as “chemokine-mediated signaling” (**Figure 5C**), cellular components including “collagen trimer complexes” (**Figure 5D**), and molecular functions like “chemokine activity” and “receptor binding” (**Figure 5E**). KEGG pathway analysis further highlighted associations with “Chemokine signaling pathway” and “Viral protein-cytokine receptor interactions” (**Figure 5F**). These findings collectively implicate chemokine-driven inflammatory responses and extracellular matrix remodeling as pivotal mechanisms linking T2DM to CAVD progression.

### 3.6 Screening of small-molecule compounds with therapeutic potential for CAVD

**Figure 6:**
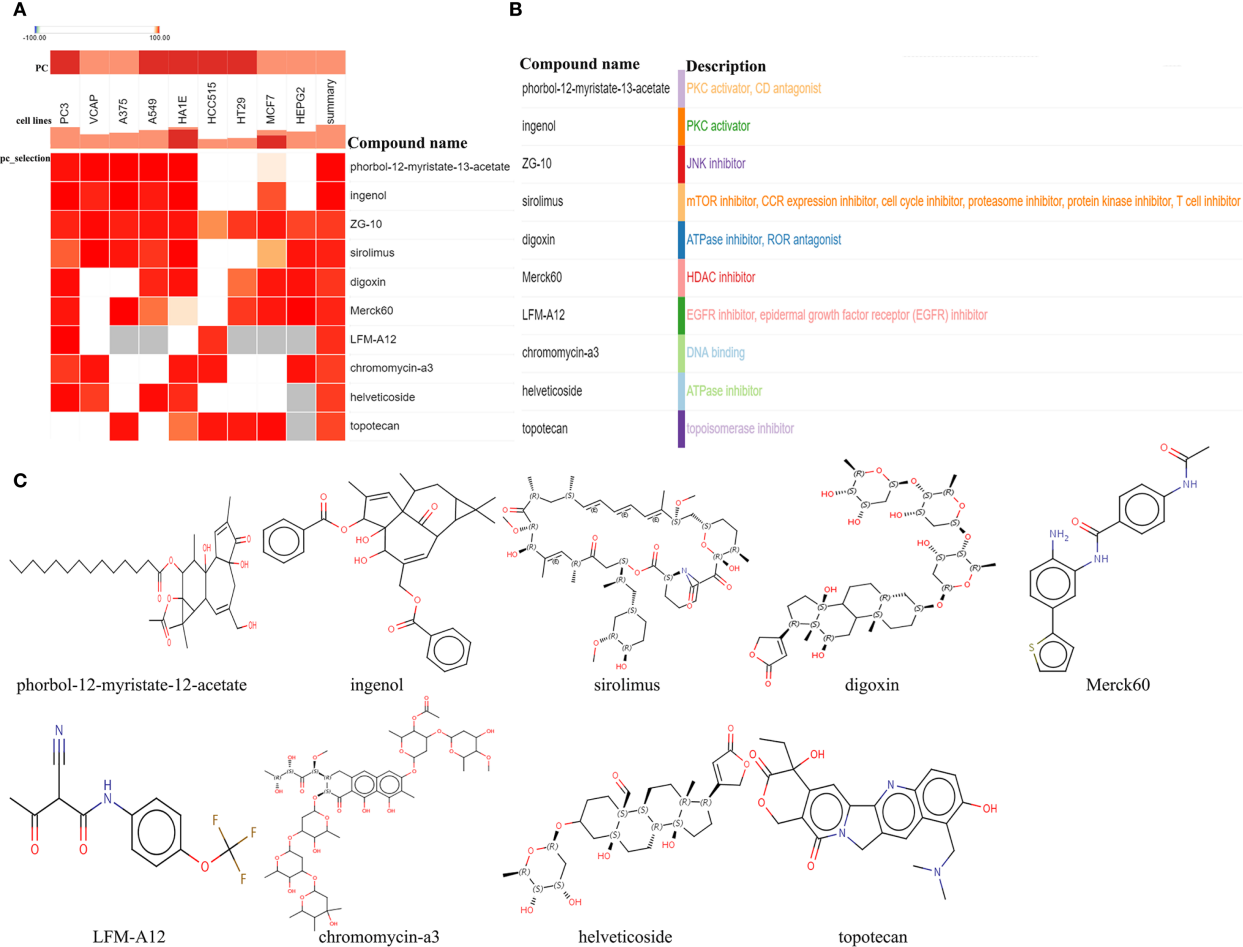
Screening of the potential small-molecular compounds for the treatment of CAVD via cMAP analysis. **(A)** The heatmap presenting the top10 compounds with the most significantly negative enrichment scores in 10 cell lines based on cMAP analysis. **(B)** The description of those top10 compounds. **(C)** The chemical structures of those 10 compounds were shown. cMAP connectivity map.

To investigate potential therapeutic agents for T2DM-associated CAVD, upregulated genes from T2DM-related pathogenic modules were analyzed using the cMAP database to identify compounds capable of reversing disease-associated transcriptional alterations. Computational screening revealed 10 top candidate compounds with the most significant negative enrichment scores (indicating reversal of disease gene expression patterns): phorbol-12-myristate-12-acetate, ingenol, ZG-10, sirolimus, digoxin, Merck60, LFM-A12, chromomycin-A3, helveticoside, and topotecan (**Figures 6A, B**). Structural motifs and predicted target pathways of these candidates—spanning immunomodulatory, epigenetic, and metabolic regulators—are systematically annotated in **Figure 6C**.

### 3.7 Development of a diagnostic model based on a machine learning–integrated framework

**Figure 7:**
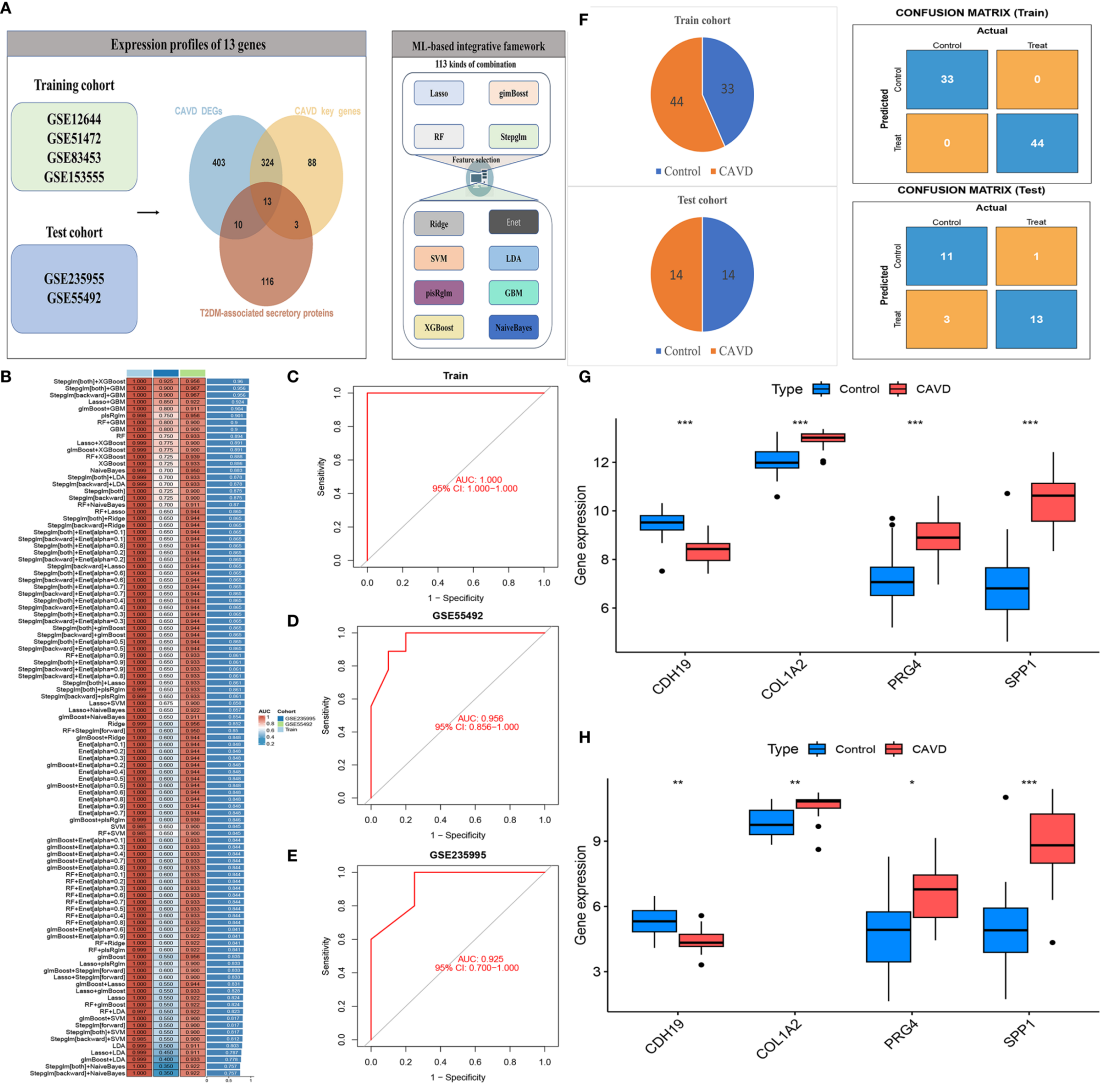
Construction of a diagnostic model for CAVD using an integrated machine learning (ML) framework. (A) Schematic of the diagnostic model built based on 13 intersecting genes (from CAVD DEGs, CAVD pathogenesis-related key genes, and T2DM-associated secreted protein DEGs). (B) Performance comparison of 113 combinatorial ML models; the optimal combo (Stepglm, bidirectional, XGBoost) achieved an average AUC of 0.95. (C–E) Visualization of the integrated ML model development process. (F) Case distribution and confusion matrices of the four-gene (CDH19, COL1A2, PRG4, SPP1) model in training and validation cohorts. (G–H) Gene expression patterns: COL1A2, PRG4, SPP1 upregulated and CDH19 downregulated in CAVD tissues vs controls.

A diagnostic model was constructed using an integrated machine learning (ML) framework, based on 13 intersecting genes identified from DEGs in CAVD, key genes implicated in CAVD pathogenesis, and secreted protein DEGs associated with T2DM (**Figure 7A**). The merged datasets from GSE12644, GSE51472, GSE153555, and GSE83453 were utilized as the training cohort for model development, while GSE235995 and GSE55492 were employed as independent validation cohorts. To build the model, we applied a comprehensive ML-based computational framework to the expression profiles of the 13 candidate genes. A total of 12 distinct ML algorithms were employed, resulting in 113 combinatorial models being evaluated. As illustrated in **Figure 7B**, the optimal combination comprising Stepglm, bidirectional and XGBoost achieved the highest average area under the curve (AUC) of 0.95 across five datasets, and was designated the optimal diagnostic model. The stacking ensemble model achieved the highest performance across multiple metrics, including Accuracy (0.94), Precision/Recall (0.98), F1 score (0.89), and Matthews Correlation Coefficient (0.89), Sensitivity/Specificity(0.98),demonstrating robust predictive capability and balance between sensitivity and specificity (**Supplementary Table S1**). The development process is depicted in **Figures 7C–E**. Based on this optimal ML combination, we constructed a diagnostic model using four hub genes(CDH19, COL1A2, PRG4, and SPP1). All of which demonstrated robust diagnostic performance, as evidenced by consistently high AUC values. This four-gene model was subsequently applied to predict disease probability in both the training and validation cohorts, yielding accurate and consistent predictive outcomes. The distribution of cases and corresponding confusion matrices for both cohorts are presented in **Figure 7F**. Furthermore, analysis of gene expression patterns between CAVD and control samples revealed upregulation of COL1A2, PRG4, and SPP1, and downregulation of CDH19 in CAVD tissues (**Figures 7G, H**).

### 3.8 Immune infiltration landscape analysis

**Figure 8:**
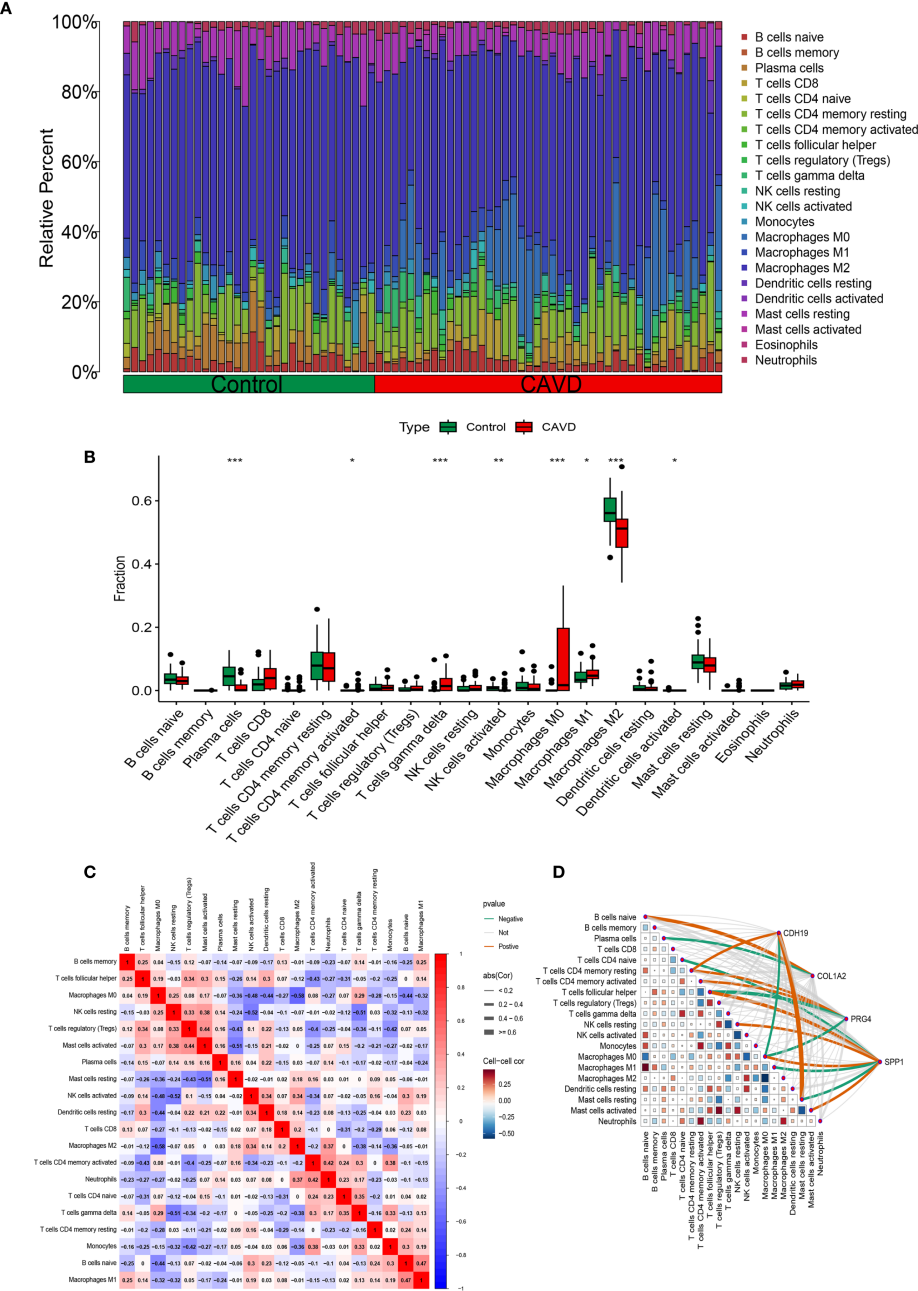
Immune infiltration analysis. **(A)** Histogram of different immune cell content in each sample. **(B)** Comparison of the content of different immune cells in normal and CAVD groups. **(C)** Correlation heatmap of all immune infiltrating cells. **(D)** Correlation network heatmap of hub genes and immune infiltrating cells.

Functional and pathway enrichment analyses of T2DM-related pathogenic genes in CAVD revealed strong associations with inflammatory and immune regulatory processes. To investigate the immune microenvironment and its relationship with diagnostic biomarkers in CAVD, we employed the CIBERSORT algorithm to estimate the composition of immune cell populations. This analysis quantified the proportions of 22 immune cell types in each sample and identified significant differences in eight immune cell subsets between calcified and control aortic valve tissues. Compared with controls, CAVD samples exhibited increased proportions of M0 macrophages, M1 macrophages, and T cells gamma delta, while showing decreased proportions of plasma cells, activated NK cells, and M2 macrophages (**Figures 8A, B**). Moreover, correlation analysis among the 22 immune cell types revealed a significant positive correlation between M1 macrophages and naïve B cells (r = 0.47, p < 0.05), as well as a significant negative correlation between M2 macrophages and M0 macrophages (r = −0.58, p < 0.05) (**Figure 8C**). Further correlation analysis between these immune cell subsets and the four identified hub genes demonstrated strong associations across all gene–immune cell pairs, with CDH19 showing a particularly strong positive correlation with Mast cell resting (**Figure 8D**).

### 3.9 Correlation analysis between immune cell infiltration and hub genes

**Figure 9:**
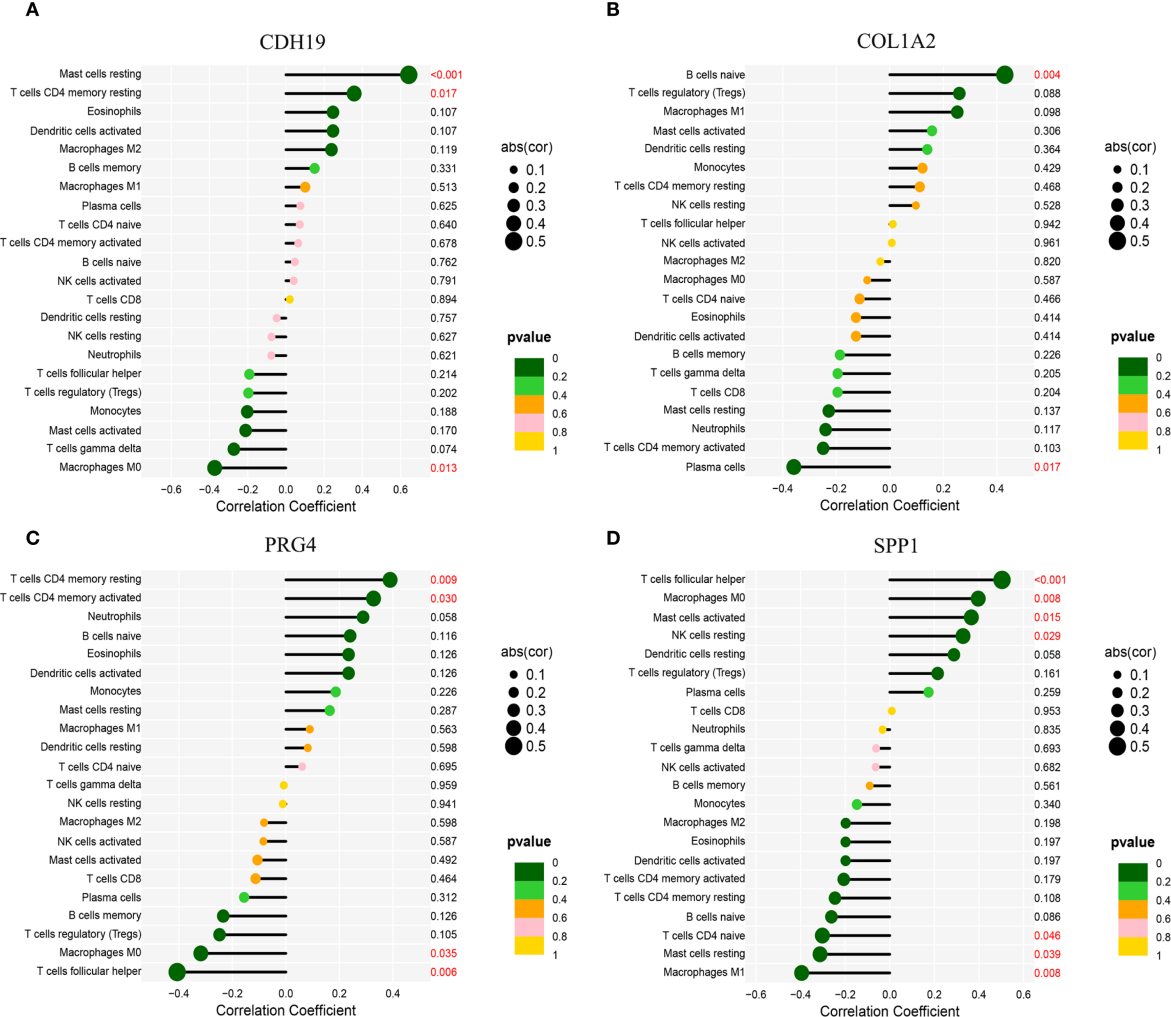
Correlation analysis between hub genes and immune cell infiltration. **(A)** Correlation of CDH19 with immune cell infiltration. **(B)** Correlation of COL1A2 with immune cell infiltration. **(C)** Correlation of PRG4 with immune cell infiltration. **(D)** Correlation of SPP1 with immune cell infiltration.*Statistical significance: *p < 0.05, **p < 0.01, **p < 0.001.

To further elucidate the expression patterns of diagnostic biomarkers and their potential associations with infiltrating immune cells, we performed a comprehensive correlation analysis (**Supplementary Figure S1**). CDH19 expression exhibited a strong positive correlation with Mast cells resting (r = 0.64, p < 0.001) and T cells CD4 memory resting (r = 0.36, p = 0.017), while displaying a negative correlation with Macrophages M0 (r = −0.37, p = 0.013) (**Figure 9A**). COL1A2 was positively correlated with B cells naive (r = 0.43, p = 0.004) and negatively correlated with Plasma cells (r = −0.36, p = 0.017) (**Figure 9B**). PRG4 demonstrated positive correlations with both T cells CD4 memory resting (r = 0.39, p = 0.009) and T cells CD4 memory activated (r = 0.33, p = 0.030), while negatively correlated with Macrophages M0 (r = −0.32, p = 0.035) (**Figure 9C**). SPP1 exhibited significant correlations with various immune cell subsets, showing positive associations with T cells follicular helper (r = 0.50, p < 0.001), Macrophages M0 (r = 0.40, p = 0.008), Mast cells activated (r = 0.37, p = 0.015), and NK cells resting (r = 0.33, p = 0.029), while demonstrating negative correlations with Macrophages M1 (r = −0.40, p = 0.008), Mast cells resting (r = −0.31, p = 0.039), and T cells CD4 naive (r = −0.30, p = 0.046) (**Figure 9D**).

### 3.10 Validation of hub gene expression in CAVD tissues with coexisting T2DM

**Figure 10:**
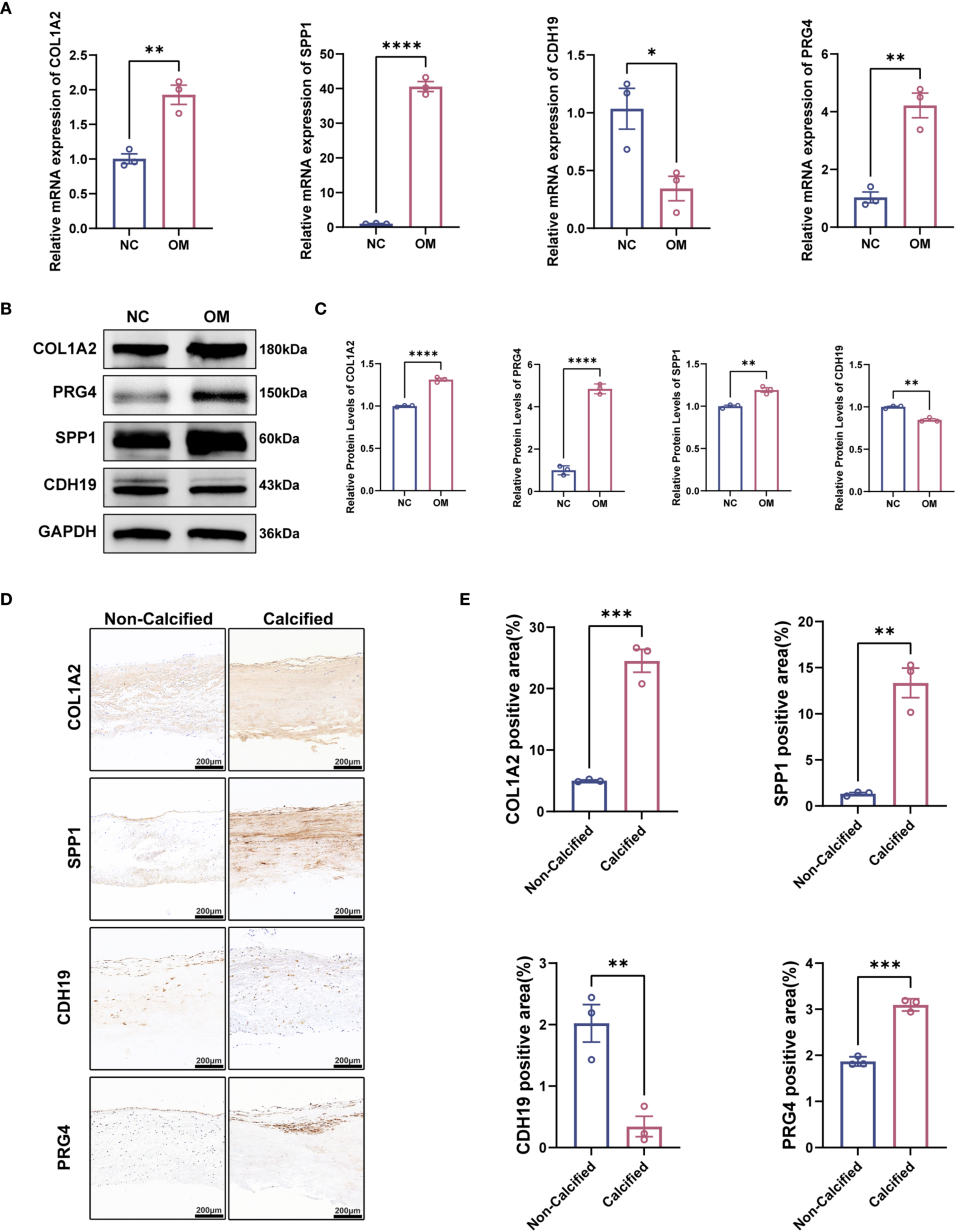
Validation of hub gene expression patterns in calcified aortic valves with T2DM. **(A)** mRNA expression levels of hub genes detected by qPCR (n = 3 per group). **(B, C)** Representative Western blot (WB) analysis and quantification of COL1A2, SPP, PRG4, and CDH19 protein levels in calcified aortic valves with T2DM (n = 3 per group). **(D)** Immunofluorescence staining of COL1A2, SPP, PRG4 and CDH19 in calcified aortic valves with T2DM. **(E)** Quantitative analysis of immunofluorescence intensity. Scale bar: 200 μm. Statistical significance was determined by two-tailed unpaired Student’s t-test. *p < 0.05, **p < 0.01, ***p < 0.001, ****p < 0.0001.

To characterize the expression profiles of CDH19, COL1A2, PRG4, and SPP1, we performed RT-qPCR and Western blot analyses on clinical tissue samples. Primers are shown in **Table 2**. Compared with normal tissues, mRNA levels of CDH19 were significantly downregulated, whereas COL1A2, PRG4, and SPP1 were markedly upregulated in CAVD tissues complicated with T2DM (**Figures 10A–D**). To corroborate these findings, Western blotting was conducted to assess the corresponding protein levels, which revealed expression patterns consistent with the mRNA results (**Figures 10B, C**). Furthermore, immunohistochemical staining was performed on human aortic valve samples obtained from Zhongnan Hospital of Wuhan University to evaluate the tissue localization and expression of CDH19, COL1A2, SPP1 and PRG4 (**Figures 10D, E**). Furthermore, to explore the potential pathways involving these hub genes, we assessed the protein expression of CXCL12 and MMP9, which were central to the bioinformatically-predicted chemokine and extracellular matrix remodeling pathways. Western blot analysis revealed a significant upregulation of MMP9 and a strong increasing trend for CXCL12 in the T2DM-CAVD group compared to controls (**Supplementary Figure S2**), providing preliminary protein-level evidence supporting the involvement of this predicted network.

**Table 2:** Primers for RT-qPCR experiments with 4 Hub Genes.

Gene id	Forward primer	Reverse primer
COL1A2	GAGGAGAGCCTGGCAACATT	AGGACCAGGGAGACCAAACT
SPP1	AATCTCCTAGCCCCACAGACC	CCACACTATCACCTCGGCCA
PRG4	CGACGCCCAATGTAAGAAGTATG	TGATGGTTTGAGATGCTCCTGAA
CDH19	CTGACGATCCCTCAAGTGGTAAT	ACCCAATACTCATCTTGCAGTTCT

RT-qPCR Reverse transcription quantitative-polymerase chain reaction.

## 4 Discussion

In recent decades, the incidence of CAVD has risen sharply. Despite this growing prevalence, effective pharmacological treatments for CAVD remain lacking, underscoring the urgent need to identify potential therapeutic options. CAVD often presents no significant symptoms in its early stages; however, once symptoms manifest, the disease is typically in its advanced stages. At this point, aortic valve replacement—whether performed through surgical or transcatheter procedures—becomes the only viable therapeutic approach (3–5). The pathological link between CAVD and T2DM has become a focal point of research in the cardiovascular and metabolic domains. While the hypothesis that T2DM accelerates CAVD progression through mechanisms such as chronic inflammation, oxidative stress, and immune dysregulation is widely accepted, the molecular mechanisms that bridge these two conditions remain inadequately understood. Therefore, the objective of our study was to utilize bioinformatics approaches to identify novel biomarkers associated with both CAVD and T2DM, as well as to screen for small molecules with potential therapeutic properties (11).

In recent years, significant breakthroughs have been made in the discovery of small molecules with therapeutic potential for a variety of diseases. Small molecules with high tissue permeability, tunable half-lives, and favorable oral bioavailability have shown great promise in therapeutic applications. For example, fluoridated enzyme inhibitors have shown significant effects in preventing calcification in CAVD (26). Additionally, the role of statins is currently under investigation, with evidence suggesting that they may help slow the pathological progression of CAVD (27). However, the development of potential therapeutic agents for CAVD requires further high-throughput screening based on gene expression profiles in calcified aortic valves, to identify more small molecules with potential efficacy. In this context, the current study, through cMAP analysis, offers a novel perspective by linking T2DM-related pathogenic genes to identify potential compounds for CAVD treatment. By applying upregulated T2DM-related pathogenic genes from calcified valves to cMAP analysis, ten small molecules (PMA, Ingenol, ZG-10, Sirolimus, Digoxin, Merck60, LFM-A12, Chromomycin-A3, Helveticoside, and Topotecan) were selected as candidate compounds. Notably, PMA (28), an effective PKC activator, exhibited the highest negative enrichment score in the cMAP analysis, suggesting its potential to reverse the upregulation of T2DM-related pathogenic genes in CAVD. Although a direct link between PMA and calcification has not yet been established, PMA has shown significant signaling modulation effects in diabetic complications (29). For example, PMA can improve cardiovascular function by inhibiting PKC-related inflammatory responses, reducing the expression of adhesion molecules (30), and suppressing monocyte accumulation. Previous studies have indicated that there is a complex relationship between T2DM and CAVD, with insulin resistance and inflammation accelerating the progression of CAVD (31), while PMA may slow the pathological development of CAVD by intervening in these key pathways. Furthermore, PMA has demonstrated its ability to modulate immune and inflammatory responses in other metabolism-related cardiovascular diseases (32, 33). Therefore, PMA represents a promising therapeutic option that may have a beneficial impact on the progression of CAVD in T2DM patients. Early intervention with PMA in T2DM patients may improve glucose metabolism and delay the progression of aortic valve calcification, thus improving patient survival and quality of life.

This study is the first to systematically analyze the molecular network driving CAVD in T2DM from the perspective of secreted proteins. By analyzing transcriptomic data from the GEO database for CAVD and T2DM, we identified a significant overlap between the 142 aberrantly expressed secreted protein genes in T2DM patients and the core genes associated with CAVD. Notably, an interaction network of 13 key genes was found to dominate valve pathology through chemokine signaling pathways and collagen remodeling mechanisms. Of particular importance, the diagnostic model constructed using machine learning further narrowed the focus to four pivotal genes CDH19, COL1A2, PRG4, and SPP1 whose diagnostic efficacy (AUC = 0.95) was validated in an independent cohort. The diagnostic model developed in this study was capable of effectively distinguishing between CAVD patients and controls, providing valuable guidance for clinical treatment. Furthermore, histological experiments confirmed the differential expression patterns of these genes in T2DM patients with CAVD. Importantly, these four hub genes (CDH19, COL1A2, PRG4, SPP1) have been identified as regulators of the cell cycle in multiple disease contexts, playing critical roles in disease pathogenesis. CDH19, a cadherin family member, regulates endothelial integrity and inflammatory responses. Its downregulation in CAVD tissues correlates with increased M0 and S100A8/A9 pathway activation, suggesting a role in mitigating immune-mediated calcification. COL1A2, a key component of type I collagen, is upregulated via hyperglycemia-induced TGF-β signaling, driving vascular smooth muscle cell transdifferentiation and fibrosis (34). Elevated COL1A2 levels correlate with B cell infiltration and collagen deposition, forming a “fibrosis-inflammation” axis (35). PRG4, an anti-inflammatory glycoprotein, is suppressed in T2DM, exacerbating TLR4/NF-κB-mediated adipose inflammation and insulin resistance (36). Its positive correlation with resting memory T cells implies a regulatory role in immune tolerance, supported by preclinical studies showing PRG4 overexpression improves glucose metabolism6 (37). SPP1 acts as a metabolic-immune hub, promoting calcification via PI3K/Akt-mediated hydroxyapatite deposition and recruiting follicular helper T cells via CXCL12-CXCR4 signaling (38, 39). Paradoxically, its glycosylation in diabetic conditions may shift its function from pro-calcific to pro-fibrotic, as observed in tumor microenvironments (40).

The innovation of this study lies in its dual breakthroughs in methodology and biological insight. By developing a cross-analysis framework between T2DM secreted proteins and CAVD genes. Our functional enrichment analysis, combined with the protein validation of CXCL12, COL1A2, and MMP9, leads us to hypothesize that a potential interaction between chemokine signaling (CXCL12) and matrix remodeling (COL1A2, MMP9) may represent a novel mechanism linking T2DM to CAVD progression. To our knowledge, this is the first study to bioinformatically predict and provide preliminary protein evidence for this specific interaction network in the context of T2DM-associated CAVD. The model optimization strategy, based on 113 machine learning combinations, not only enhances diagnostic efficacy, but also identifies non-classical biomarkers, such as CDH19, providing new targets for liquid biopsy development. Immune microenvironment analysis reveals an imbalance in the M1/M2 macrophage ratio and an expansion of T cells CD4 memory resting, providing a theoretical foundation for immunotherapy targeting immune checkpoints. Moreover, among the 10 compounds predicted by cMAP, sirolimus and topotecan have been confirmed to inhibit vascular smooth muscle cell osteogenic differentiation (41, 42), aligning closely with the mechanisms predicted in this study and highlighting the application value of bioinformatics-guided drug repositioning.

However, this study does have some limitations. Despite integrating multiple datasets and applying batch correction, the sample size may restrict the precision of the diagnostic model. It is important to note that our diagnostic model distinguishes between existing CAVD patients and controls. Its potential for predicting future disease risk (prognosis) requires validation in longitudinal prospective studies. Additionally, the lack of T2DM stratification analysis makes it difficult to distinguish the impact of blood glucose control on gene expression. While wet-lab experiments validated the gene expression trends, further studies and clinical trials are required to elucidate the specific role of key genes in valve cells and confirm our findings.

## 5 Conclusion

We have uncovered the inflammatory immune pathways underlying T2DM-related CAVD and developed a CAVD diagnostic model based on CDH19, COL1A2, PRG4, and SPP1 using machine learning. This provides new insights for future diagnostic and therapeutic interventions based on serum for T2DM-associated CAVD.

## Data Availability

The original contributions presented in the study are included in the article/**Supplementary Material**. Further inquiries can be directed to the corresponding authors.

## Supplementary material

Supplementary Figure 1 Correlation between hub gene expression and immune infiltration assessed by CIBERSORT analysis. **(A–C)** Correlation of CDH19 expression with CIBERSORT immune scores. **(D, E)** Correlation of COL1A2A expression with CIBERSORT immune scores. **(F–I)** Correlation of PRG4 expression with CIBERSORT immune scores. **(J–P)** Correlation of SPP1 expression with CIBERSORT immune scores.

Supplementary Figure 2 mRNA and protein expression validation of CXCL12 and MMP9 in the predicted pathway. **(A)** mRNA expression levels of CXCL12 and MMP9 in control (n=3) and T2DM-CAVD (n=3) human aortic valve tissues as determined by RT-qPCR. *p < 0.05, **p < 0.01, ***p < 0.001, ****p < 0.0001, Student’s *t*-test).**(B)** Representative Western blot images showing protein levels of CXCL12 and MMP9. **(C)** Quantitative analysis of CXCL12 and MMP9 protein expression levels from (B). Data are presented as mean ± SEM (n = 3 per group). (*p < 0.05, **p < 0.01, ***p < 0.001, ****p < 0.0001, ns: not significant, Student’s *t*-test).

Supplementary Table 1 Comprehensive performance evaluation of the optimal stacking ensemble model.
