# Supplementary figures and images for: Integrating bioinformatics and machine learning analyses to identify immune-related secretory proteins and therapeutic small-molecule drugs in calcific aortic valve disease with type 2 diabetes
Source: Front Immunol. 2025 Oct 8;16:1634655. doi: 10.3389/fimmu.2025.1634655 (PMC12540134; doi:10.3389/fimmu.2025.1634655)

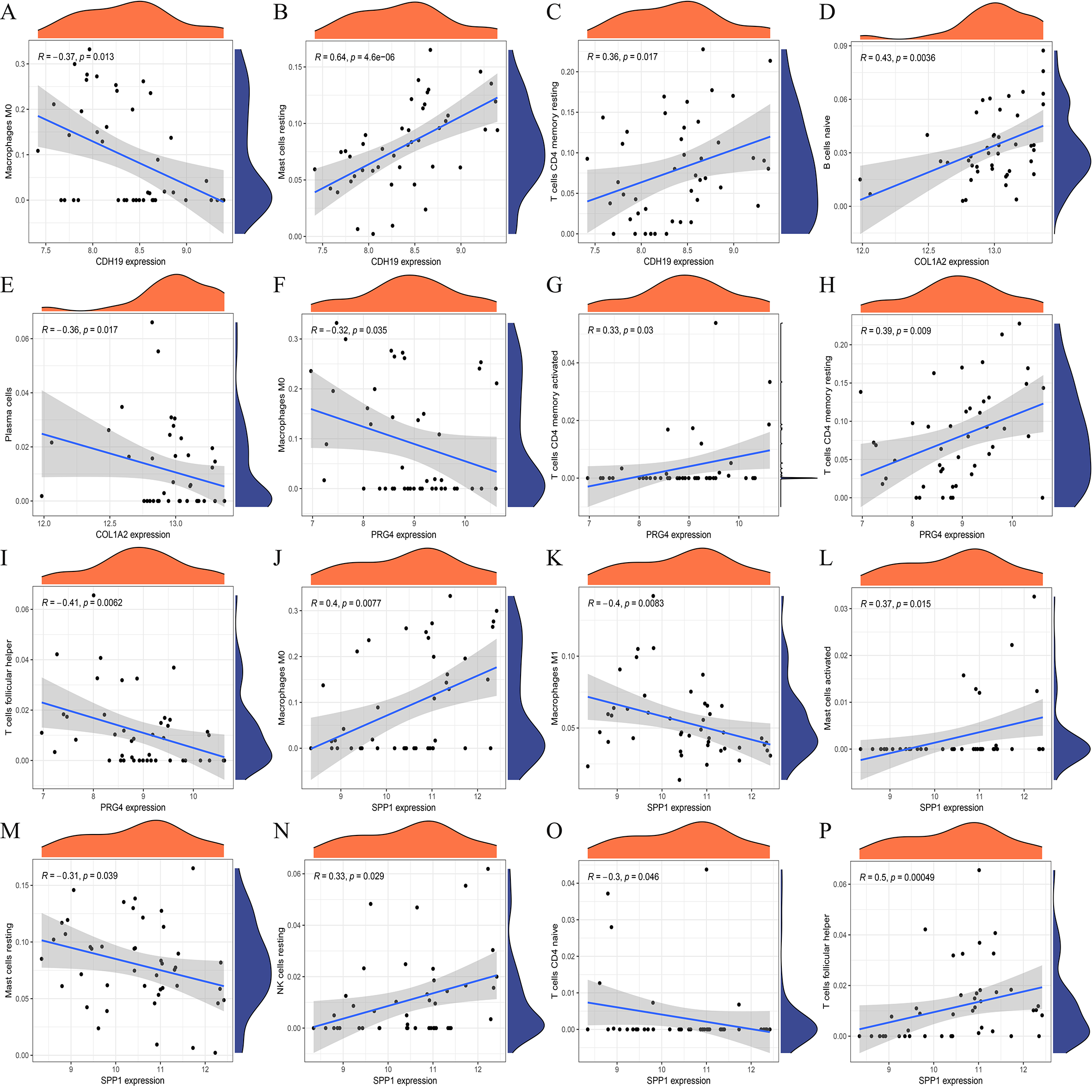

Supplement: Supplementary Figure 1 — Correlation between hub gene expression and immune infiltration assessed by CIBERSORT analysis. (A–C) Correlation of CDH19 expression with CIBERSORT immune scores. (D, E) Correlation of COL1A2A expression with CIBERSORT immune scores. (F–I) Correlation of PRG4 expression with CIBERSORT immune scores. (J–P) Correlation of SPP1 expression with CIBERSORT immune scores. [file Image1.tif]

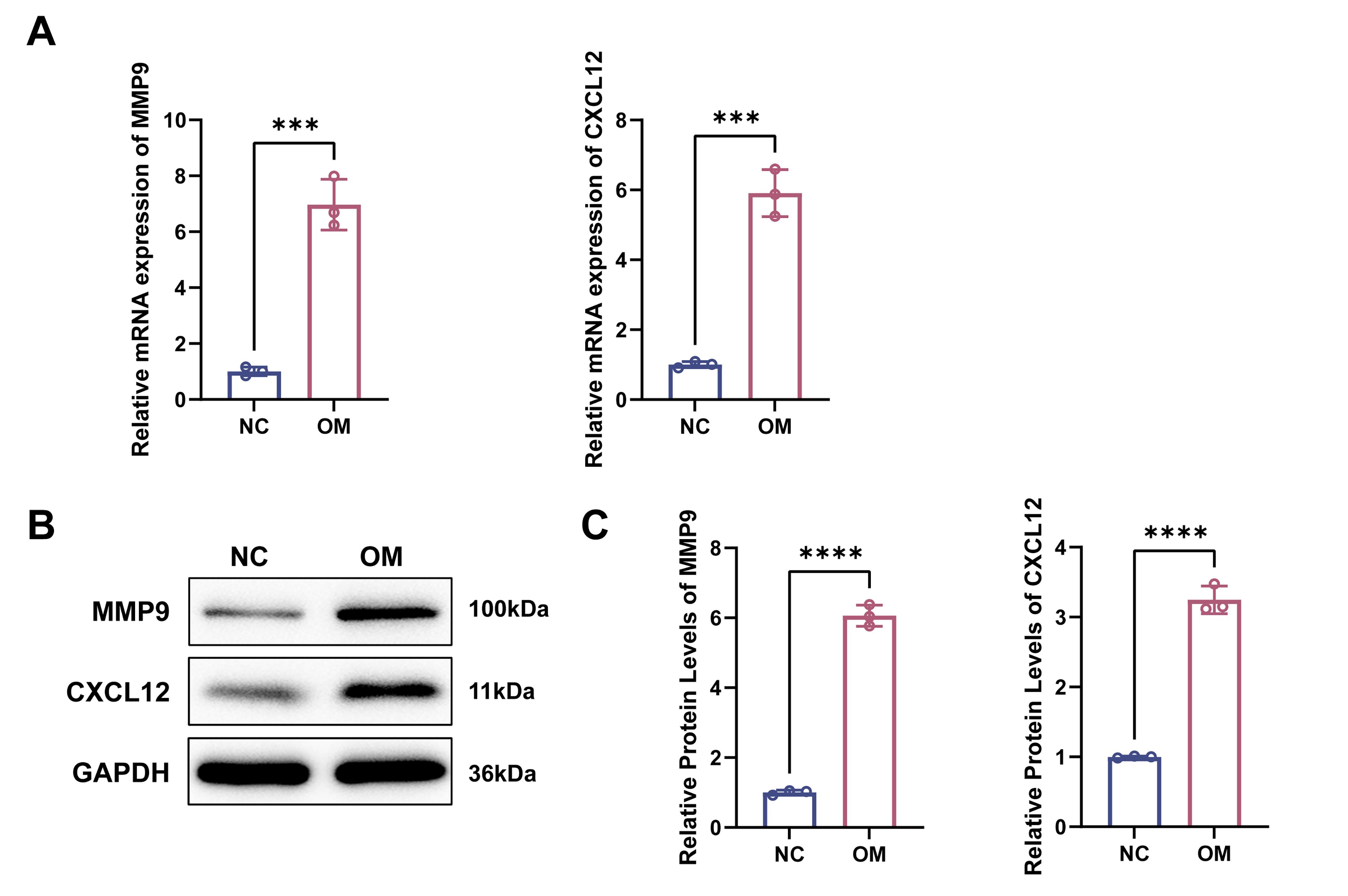

Supplement: Supplementary Figure 2 — mRNA and protein expression validation of CXCL12 and MMP9 in the predicted pathway. (A) mRNA expression levels of CXCL12 and MMP9 in control (n=3) and T2DM-CAVD (n=3) human aortic valve tissues as determined by RT-qPCR. *p < 0.05, **p < 0.01, ***p < 0.001, ****p < 0.0001, Student’s *t*-test).(B) Representative Western blot images showing protein levels of CXCL12 and MMP9. (C) Quantitative analysis of CXCL12 and MMP9 protein expression levels from (B). Data are presented as mean ± SEM (n = 3 per group). (*p < 0.05, **p < 0.01, ***p < 0.001, ****p < 0.0001, ns: not significant, Student’s *t*-test). [file Image2.tif]
