# Supplementary material for: Integrating bioinformatics and machine learning analyses to identify immune-related secretory proteins and therapeutic small-molecule drugs in calcific aortic valve disease with type 2 diabetes
Source: Front Immunol. 2025 Oct 8;16:1634655. doi: 10.3389/fimmu.2025.1634655 (PMC12540134; doi:10.3389/fimmu.2025.1634655)
Supplement: Supplementary Table 1 — Comprehensive performance evaluation of the optimal stacking ensemble model. [file Table1.docx]

| **Table S1** Comprehensive performance evaluation of the optimal stacking ensemble model | | | | |  |  |
| --- | --- | --- | --- | --- | --- | --- |
| **Method** | **Accuracy** | **Precision / Recall** | **F1 score** | **Matthews Correlation Coefficient** | **Sensitivity / Specificity** |  |
|  |  |  |  |  |  |  |
| Stepglm[both]+XGBoost | 0.94 | 0.98 | 0.89 | 0.89 | 0.98 |  |
| Stepglm[both]+GBM | 0.94 | 0.96 | 0.91 | 0.91 | 0.97 |  |
| Stepglm[backward]+GBM | 0.94 | 0.98 | 0.89 | 0.89 | 0.98 |  |
| Lasso+GBM | 0.95 | 0.96 | 0.94 | 0.94 | 0.97 |  |
| glmBoost+GBM | 0.95 | 0.98 | 0.91 | 0.91 | 0.98 |  |
| plsRglm | 0.94 | 0.96 | 0.91 | 0.91 | 0.97 |  |
| RF+GBM | 0.95 | 0.98 | 0.91 | 0.91 | 0.98 |  |
| GBM | 0.94 | 0.96 | 0.91 | 0.91 | 0.97 |  |
| RF | 0.94 | 0.96 | 0.91 | 0.91 | 0.97 |  |
| Lasso+XGBoost | 0.94 | 0.96 | 0.91 | 0.91 | 0.97 |  |
| glmBoost+XGBoost | 0.95 | 0.98 | 0.91 | 0.91 | 0.98 |  |
| RF+XGBoost | 0.95 | 0.98 | 0.91 | 0.91 | 0.98 |  |
| XGBoost | 0.94 | 0.96 | 0.91 | 0.91 | 0.97 |  |
| NaiveBayes | 0.95 | 0.98 | 0.91 | 0.91 | 0.98 |  |
| Stepglm[both]+LDA | 0.94 | 0.96 | 0.91 | 0.91 | 0.97 |  |
| Stepglm[backward]+LDA | 0.95 | 0.98 | 0.91 | 0.91 | 0.98 |  |
| Stepglm[both] | 0.95 | 0.98 | 0.91 | 0.91 | 0.98 |  |
| Stepglm[backward] | 0.94 | 0.96 | 0.91 | 0.91 | 0.97 |  |
| RF+NaiveBayes | 0.94 | 0.96 | 0.91 | 0.91 | 0.97 |  |
| RF+Lasso | 0.94 | 0.96 | 0.91 | 0.91 | 0.97 |  |
| Stepglm[both]+Ridge | 0.94 | 0.96 | 0.91 | 0.91 | 0.97 |  |
| Stepglm[backward]+Ridge | 0.94 | 0.96 | 0.91 | 0.91 | 0.97 |  |
| Stepglm[both]+Enet[alpha=0.1] | 0.94 | 0.96 | 0.91 | 0.91 | 0.97 |  |
| Stepglm[backward]+Enet[alpha=0.1] | 0.94 | 0.96 | 0.91 | 0.91 | 0.97 |  |
| Stepglm[both]+Enet[alpha=0.8] | 0.94 | 0.96 | 0.91 | 0.91 | 0.97 |  |
| Stepglm[both]+Enet[alpha=0.2] | 0.94 | 0.96 | 0.91 | 0.91 | 0.97 |  |
| Stepglm[backward]+Enet[alpha=0.2] | 0.94 | 0.96 | 0.91 | 0.91 | 0.97 |  |
| Stepglm[backward]+Lasso | 0.95 | 0.98 | 0.91 | 0.91 | 0.98 |  |
| Stepglm[both]+Enet[alpha=0.6] | 0.94 | 0.96 | 0.91 | 0.91 | 0.97 |  |
| Stepglm[backward]+Enet[alpha=0.6] | 0.94 | 0.96 | 0.91 | 0.91 | 0.97 |  |
| Stepglm[both]+Enet[alpha=0.7] | 0.94 | 0.96 | 0.91 | 0.91 | 0.97 |  |
| Stepglm[backward]+Enet[alpha=0.7] | 0.94 | 0.98 | 0.89 | 0.89 | 0.98 |  |
| Stepglm[both]+Enet[alpha=0.4] | 0.94 | 0.96 | 0.91 | 0.91 | 0.97 |  |
| Stepglm[backward]+Enet[alpha=0.4] | 0.94 | 0.96 | 0.91 | 0.91 | 0.97 |  |
| Stepglm[both]+Enet[alpha=0.3] | 0.94 | 0.98 | 0.89 | 0.89 | 0.98 |  |
| Stepglm[backward]+Enet[alpha=0.3] | 0.95 | 0.98 | 0.91 | 0.91 | 0.98 |  |
| Stepglm[both]+glmBoost | 0.93 | 0.95 | 0.89 | 0.89 | 0.97 |  |
| Stepglm[backward]+glmBoost | 0.94 | 0.96 | 0.91 | 0.91 | 0.97 |  |
| Stepglm[both]+Enet[alpha=0.5] | 0.95 | 0.98 | 0.91 | 0.91 | 0.98 |  |
| Stepglm[backward]+Enet[alpha=0.5] | 0.95 | 0.98 | 0.91 | 0.91 | 0.98 |  |
| RF+Enet[alpha=0.9] | 0.95 | 0.98 | 0.91 | 0.91 | 0.98 |  |
| Stepglm[both]+Enet[alpha=0.9] | 0.95 | 0.98 | 0.91 | 0.91 | 0.98 |  |
| Stepglm[backward]+Enet[alpha=0.9] | 0.95 | 0.98 | 0.91 | 0.91 | 0.98 |  |
| Stepglm[backward]+Enet[alpha=0.8] | 0.95 | 0.98 | 0.91 | 0.91 | 0.98 |  |
| Stepglm[both]+Lasso | 0.94 | 0.98 | 0.89 | 0.89 | 0.98 |  |
| Stepglm[both]+plsRglm | 0.95 | 0.98 | 0.91 | 0.91 | 0.98 |  |
| Stepglm[backward]+plsRglm | 0.95 | 0.98 | 0.91 | 0.91 | 0.98 |  |
| Lasso+SVM | 0.95 | 0.98 | 0.91 | 0.91 | 0.98 |  |
| Lasso+NaiveBayes | 0.96 | 0.98 | 0.94 | 0.94 | 0.98 |  |
| glmBoost+NaiveBayes | 0.96 | 0.98 | 0.94 | 0.94 | 0.98 |  |
| Ridge | 0.96 | 0.98 | 0.94 | 0.94 | 0.98 |  |
| RF+Stepglm[forward] | 0.96 | 0.98 | 0.94 | 0.94 | 0.98 |  |
| glmBoost+Ridge | 0.94 | 0.96 | 0.91 | 0.91 | 0.97 |  |
| Enet[alpha=0.1] | 0.94 | 0.96 | 0.91 | 0.91 | 0.97 |  |
| Enet[alpha=0.2] | 0.93 | 0.95 | 0.89 | 0.89 | 0.97 |  |
| Enet[alpha=0.3] | 0.93 | 0.93 | 0.91 | 0.91 | 0.95 |  |
| glmBoost+Enet[alpha=0.2] | 0.96 | 0.98 | 0.94 | 0.94 | 0.98 |  |
| Enet[alpha=0.4] | 0.95 | 0.96 | 0.94 | 0.94 | 0.97 |  |
| Enet[alpha=0.5] | 0.95 | 0.96 | 0.94 | 0.94 | 0.97 |  |
| glmBoost+Enet[alpha=0.5] | 0.96 | 0.98 | 0.94 | 0.94 | 0.98 |  |
| Enet[alpha=0.6] | 0.94 | 0.94 | 0.94 | 0.94 | 0.95 |  |
| Enet[alpha=0.8] | 0.94 | 0.96 | 0.91 | 0.91 | 0.97 |  |
| Enet[alpha=0.9] | 0.93 | 0.93 | 0.91 | 0.91 | 0.95 |  |
| Enet[alpha=0.7] | 0.94 | 0.98 | 0.89 | 0.89 | 0.98 |  |
| glmBoost+plsRglm | 0.92 | 0.94 | 0.90 | 0.90 | 0.95 |  |
| SVM | 0.92 | 0.94 | 0.90 | 0.90 | 0.95 |  |
| RF+SVM | 0.92 | 0.94 | 0.90 | 0.90 | 0.95 |  |
| glmBoost+Enet[alpha=0.1] | 0.92 | 0.94 | 0.90 | 0.90 | 0.95 |  |
| glmBoost+Enet[alpha=0.3] | 0.92 | 0.94 | 0.90 | 0.90 | 0.95 |  |
| glmBoost+Enet[alpha=0.4] | 0.93 | 0.96 | 0.90 | 0.90 | 0.96 |  |
| glmBoost+Enet[alpha=0.7] | 0.92 | 0.94 | 0.90 | 0.90 | 0.95 |  |
| glmBoost+Enet[alpha=0.8] | 0.92 | 0.94 | 0.90 | 0.90 | 0.95 |  |
| RF+Enet[alpha=0.1] | 0.92 | 0.94 | 0.90 | 0.90 | 0.95 |  |
| RF+Enet[alpha=0.2] | 0.92 | 0.96 | 0.88 | 0.88 | 0.96 |  |
| RF+Enet[alpha=0.3] | 0.92 | 0.94 | 0.90 | 0.90 | 0.95 |  |
| RF+Enet[alpha=0.6] | 0.92 | 0.94 | 0.90 | 0.90 | 0.95 |  |
| RF+Enet[alpha=0.7] | 0.92 | 0.96 | 0.88 | 0.88 | 0.96 |  |
| RF+Enet[alpha=0.5] | 0.93 | 0.96 | 0.90 | 0.90 | 0.96 |  |
| RF+Enet[alpha=0.4] | 0.91 | 0.94 | 0.88 | 0.88 | 0.95 |  |
| RF+Enet[alpha=0.8] | 0.92 | 0.94 | 0.90 | 0.90 | 0.95 |  |
| glmBoost+Enet[alpha=0.6] | 0.93 | 0.96 | 0.90 | 0.90 | 0.96 |  |
| glmBoost+Enet[alpha=0.9] | 0.93 | 0.96 | 0.90 | 0.90 | 0.96 |  |
| RF+Ridge | 0.93 | 0.96 | 0.90 | 0.90 | 0.96 |  |
| RF+plsRglm | 0.93 | 0.96 | 0.90 | 0.90 | 0.96 |  |
| glmBoost | 0.93 | 0.96 | 0.90 | 0.90 | 0.96 |  |
| Lasso+plsRglm | 0.93 | 0.96 | 0.90 | 0.90 | 0.96 |  |
| glmBoost+Stepglm[forward] | 0.92 | 0.96 | 0.88 | 0.88 | 0.96 |  |
| Lasso+Stepglm[forward] | 0.93 | 0.96 | 0.90 | 0.90 | 0.96 |  |
| glmBoost+Lasso | 0.93 | 0.96 | 0.90 | 0.90 | 0.96 |  |
| Lasso+glmBoost | 0.93 | 0.96 | 0.90 | 0.90 | 0.96 |  |
| Lasso | 0.94 | 0.96 | 0.92 | 0.92 | 0.96 |  |
| RF+glmBoost | 0.94 | 0.96 | 0.92 | 0.92 | 0.96 |  |
| RF+LDA | 0.94 | 0.96 | 0.92 | 0.92 | 0.96 |  |
| glmBoost+SVM | 0.94 | 0.96 | 0.92 | 0.92 | 0.96 |  |
| Stepglm[forward] | 0.92 | 0.94 | 0.90 | 0.90 | 0.95 |  |
| Stepglm[both]+SVM | 0.92 | 0.94 | 0.90 | 0.90 | 0.95 |  |
| Stepglm[backward]+SVM | 0.91 | 0.94 | 0.88 | 0.88 | 0.95 |  |
| LDA | 0.91 | 0.92 | 0.90 | 0.90 | 0.93 |  |
| Lasso+LDA | 0.94 | 0.96 | 0.92 | 0.92 | 0.96 |  |
| glmBoost+LDA | 0.93 | 0.94 | 0.92 | 0.92 | 0.95 |  |
| Stepglm[both]+NaiveBayes | 0.93 | 0.94 | 0.92 | 0.92 | 0.95 |  |
| Stepglm[backward]+NaiveBayes | 0.94 | 0.96 | 0.92 | 0.92 | 0.96 |  |
